# Validation of the DSM-5 internet gaming disorder framework for clinical diagnosis of problematic social media usage

**DOI:** 10.1016/j.abrep.2026.100673

**Published:** 2026-02-01

**Authors:** Wenxia Xie, Shaoke Cao, Xuelin Chao, Qian Sun, Qing Zou, Chuanjian Liu, Guojiang Wu, Qiaosheng Liu, Xiaoping Wang, Wei Hao, Yanhui Liao, Tao Luo

**Affiliations:** a Department of Psychiatry, the First Affiliated Hospital of Nanchang University, Nanchang, Jiangxi 310006, China; b Department of Psychiatry, the Second Xiangya Hospital, Central South University, Changsha, Hunan 410011, China; c Department of Psychiatry, Sir Run Run Shaw Hospital, School of Medicine, Zhejiang University, Hangzhou, Zhejiang 310016, China; d Department of Psychology, Jiangxi Mental Hospital of Nanchang University, Nanchang, Jiangxi, China

**Keywords:** Problematic Social media usage (PUSM), DSM-5, ICD-11, Criteria, Diagnostic accuracy, Clinical validity

## Abstract

**Aims:**

Problematic social media usage (PUSM) has become a growing public health issue, with adolescents being particularly vulnerable. The absence of a standardized diagnostic tool has hindered consistent clinical identification and research advancement. To address this gap, the present study systematically evaluated the applicability of the DSM-5 Internet gaming disorder (IGD) criteria for diagnosing PUSM.

**Methods:**

A total of 405 participants were recruited and divided into five groups: PUSM, gaming disorder (GD), regular social media users (RSMU), regular gamers (RG), and healthy controls (HC). In this study, patients were evaluated using both ICD-11 and DSM-5. The ICD-11 criteria served as an external criterion, providing a benchmark for the validity testing of the DSM-5 framework.

**Results:**

The DSM-5 IGD criteria demonstrated excellent diagnostic accuracy (>80%) for both PUSM and GD, except “deception”. The ICD-11 and DSM-5 criteria exhibited high consistency, though the ICD-11 criteria adopted a stricter diagnostic threshold. No significant differences were observed between the PUSM and GD groups in terms of symptom profiles, functional impairment, or clinical severity.

**Discussion and conclusions:**

This study provides empirical support for adopting the DSM-5 IGD diagnostic criteria as a standardized clinical tool for assessing PUSM. However, the “deception” criterion requires further validation due to its weak diagnostic performance. The findings further confirm the conceptual and symptomatic homogeneity between PUSM and IGD, supporting their classification within a unified behavioral addiction framework.

## Highlights

• The DSM-5 IGD diagnostic criteria, except “deception,” have a diagnostic accuracy of over 80% for social media addiction.
• PUSM and GD show no significant differences in core symptoms, social impairment, and clinical severity.
• The kappa value was 0.91 between the DSM-5 and ICD-11 criteria..

## 1 Introduction

Social media, including websites and online applications, has fundamentally evolved from a simple platform for information exchange into a complex ecosystem that integrates social maintenance, personal image crafting, entertainment, and emotional support (Kietzmann et al., 2011). In China, the number of social media users has reached 1.11 billion (CINIC, 2025). Problematic Social Media Usage (PSMU) refers to individuals showing insufficient control, excessive use, or dependence when using social media, leading to impaired functionality in daily life and the generation of psychological or social problems (Fineberg et al., 2022). Given the absence of unified classification criteria and assessment tools, the estimated prevalence of problematic social media use in the general population worldwide varies significantly, ranging from 3.5% to 25% (Cheng et al., 2021, Luo et al., 2021). Concurrently, a growing body of research suggests that PSMU was associated with a range of adverse outcomes, including self-reported mental health issues (Ahmed et al., 2024), impaired academic and occupational functioning (Aslan & Polat, 2024), and suicidal behaviors Xiao and Meng, 2025). It is important to note that many of these studies are correlational, and the relationships may be influenced by or coexist with primary mental health conditions. Nonetheless, its potential impact makes it a significant medical and public health concern (Rumpf et al., 2018).

The increasing body of evidence has prompted many scholars to advocate for classifying PSMU as an independent behavioral addiction (Griffiths, 2017, Keles et al., 2020, Ryan et al., 2014, Van Den Eijnden et al., 2018). Moretta and Wegmann (2025) proposes classifying PSMU as a pathological condition, as it shares with addictive behaviors the core mechanisms of maladaptive reward activation and impaired inhibitory control, along with associated maladaptive patterns. Brand et al. (2025) emphasizes that such behavioral addictions, including PSMU, are highly prevalent and frequently co-occur with other mental disorders, yet often remain undetected, leading to poor outcomes, thus advocating for proactive screening and systematic treatment as necessary measures. Nevertheless, a significant practical obstacle remains: the lack of a universally accepted gold standard for diagnosis. Early on, scholars defined PUSM based on Griffiths’ six core components of addictive behaviors: salience, mood modification, tolerance, withdrawal symptoms, conflict, and relapse (Griffiths et al., 2016, Starcevic, 2017). In 2013, the American Psychiatric Association introduced Internet Gaming Disorder (IGD) into the DSM-5 as a condition warranting further study (American Psychiatric Association, 2013). The proposed diagnostic framework consists of nine criteria: preoccupation, withdrawal, tolerance, loss of control, loss of interest, giving up other activities, continuation despite problems, deception, and escape (American Psychiatric Association, 2013). The DSM-5 diagnostic criteria for IGD have been widely applied to the definition of problematic social media usage (van den Eijnden et al., 2016). In 2018, Gaming Disorder was officially recognized as a mental disorder in the ICD-11. Unlike the DSM-5 framework, the ICD-11 framework emphasizes: 1) impaired control over the behavior, 2) prioritization of the behavior over other interests and daily activities, and 3) continuation or escalation of the behavior despite experiencing negative consequences; 4) significant impairment in personal, family, social, educational, occupational, or other important areas of functioning (World Health Organization, 2018).

The ICD-11 diagnostic framework has been evaluated as having high diagnostic validity and clinical utility for gaming disorder (Chih-Hung Ko, 2020, Yen et al., 2022). However, the efficacy of the DSM-5′s IGD framework is controversial, with debates centered on three key areas. Firstly, the representativeness of the criteria is questioned. Concepts such as “tolerance” and “withdrawal symptoms,” which originate from the neurobiological adaptations seen in substance use disorders, may not be fully applicable to behavioral addictions that primarily involve psychological processes (Griffiths et al., 2016, Starcevic, 2017). Secondly, certain criteria have issues with discriminant validity. For example, “salience” has been criticized as being too broad, with a risk of pathologizing highly engaged but non-pathological users, such as professional esports players or enthusiastic hobbyists (Griffiths et al., 2016, Kardefelt-Winther, 2014, Kardefelt-Winther, 2015). Thirdly, cultural differences affect items like “deception”. The motivation for concealing online use may be influenced by cultural norms regarding privacy and social judgment, making it a less reliable universal indicator of pathology (Kardefelt-Winther, 2015; C. H. Ko et al., 2014). In contrast, “unsuccessful attempts to control or cut back use,” “use despite significant problems,” and “significant negative consequences” are the least controversial criteria, demonstrating strong reliability and predictive validity (Billieux et al., 2017, Carbonell, 2017, Griffiths, 2017, Griffiths et al., 2016).

However, current evidence is mainly based on cross-sectional questionnaire data. There is currently a lack of a systematic study on the diagnostic efficacy of the DSM-5′s nine diagnostic criteria in a rigorously defined clinical sample. A clinical sample is the “gold standard” for validation. In this population, symptoms are likely to be more severe, and the degree of social and occupational impairment is expected to be more significant and complex. The true diagnostic validity of any set of criteria must be tested in this real-world context.

This study aims to assess the diagnostic efficacy of the nine DSM-5-IGD criteria for PUSM through a clinical sample, in order to validate their reliability and practical value. It also further evaluate the clinical symptom homogeneity between PUSM and IGD to explore the possibility of classifying both within a unified “behavioral” addiction framework. This research bridges the gap between theory and clinical practice, aiming to provide a robust framework for identifying and treating PUSM.

Specifically, this study will focus on addressing the following key questions:

1. Given the current lack of diagnostic criteria for problematic social media usage, we employed a semi-structured clinical interview based on the ICD-11-GD framework to diagnose participants. Based on the established correlation between addiction and factors such as depression and impulsivity traits, we employed them as key external criteria (9-item Patient Health Questionnaire and the Brief Barratt Impulsiveness Scale) to test the validity of the adapted ICD-11 GD criteria in diagnosing PSMU.
2. Using clinical diagnosis based on ICD-11-GD as the gold standard, we examined the reliability, validity, and diagnostic accuracy of the DSM-5 IGD criteria in the context of social media use disorder.
3. We compared the differences between GD and PUSM in terms of symptoms, functional impairment, and clinical severity to reveal their similarities and differences within a behavioral addiction framework.

## 2 Methods

### 2.1 Participants

Participants with PUSM/GD were recruited by advertisement from individuals who visited the outpatient clinic of Jiangxi Mental Hospital and the First Affiliated Hospital of Nanchang University between August 2021 and September 2025 due to excessive Internet use. Prior to recruitment, the scope of social media was operationalized, the term encompassed major platforms prevalent in China, including but not limited to WeChat (including Moments), Weibo, Douyin (TikTok), Bilibili, QQ Zone, and Xiaohongshu. Informed by the clinical profile in China, where individuals seeking consultation for problematic internet use are predominantly adolescents, typically flagged by parental concern over prolonged engagement exceeding 40 h per week, we established the following inclusion criteria: (1) aged 12–––19 years; (2) engaged in social media/gaming for ≥ 40 h per week; and (3) a consistent pattern of social media use/gaming > 1 year; (4) a diagnosis of PUSM or GD confirmed by meeting the ICD-11 diagnostic criteria following a clinical interview.

Participants for the regular social media user (RSMU), regular gamer (RG), and healthy control (HC) groups were recruited via advertisements from a local middle school during the same period. The participants were carefully selected to ensure gender and age balance with those in the PUSM/IGD group. The following inclusion criteria were adopted for the RSMU/RG group: (1) aged 12–19 years; (2) a consistent pattern of social media use/gaming for >1 year; and (3) did not meet the diagnostic criteria for PUSM or GD. For the HC group, the following inclusion criteria were adopted: (1) aged 12–19 years; (2) did not engage in regular social media using and gaming; and (3) did not meet the diagnostic criteria for PUSM and GD.

The following exclusion criteria were applied to all groups: (1) A current diagnosis of other psychiatric disorders (e.g., major depressive disorder, bipolar I disorder, psychotic disorders, substance use disorders, and other behavioral addictions); (2) A dual diagnosis of problematic social media usage and gaming addiction; or (3) A history of brain trauma or severe physical illness.

In total, 405 participants were included, with 81 participants in each group (i.e., the PUSM group, the IGD group, the RSMU group, the RG group, and the HC group). Informed consent was obtained from all participants, while parents’ permission was also obtained for those less than 18 years of age. The procedures were carried out in accordance with the Declaration of Helsinki. Ethical approval was also attained from the local ethics committee (No. 20180112).

### 2.2 Procedure

Clinical interviews were conducted with participants and their families by a psychiatrist. When contradictory information was provided by the participant and the parent, objective data was used for determination. For example, in cases where reported social media/gaming time from the participant and the parent was inconsistent, the screen time data from the phone was referenced. Following the clinical interviews, participants completed a battery of self-report questionnaires to assess: (1) the severity of gaming disorder and Problematic Social Media Usage; (2) levels of depression; and (3) traits of impulsivity.

### 2.3 Measures

#### 2.3.1 Socio-demographics and usage patterns

Socio-demographic data, including gender and age, were collected. To estimate weekly usage time, a calculation formula was applied: (Daily use/play time on a weekday × 5) + (Daily use/play time on a weekend day × 2).

#### 2.3.2 Clinical diagnoses

##### 2.3.2.1 ICD-11 criteria for PUSM/GD

Participants were required to fulfill all four of the ICD-11 criteria for gaming disorder (applied analogously to social media use): “loss of control,” “giving up other activities,” “continuation despite problems,” and “negative consequences” (World Health Organization, 2018). This study used a semi-structured interview schedule based on Ko to assess four ICD-11 criteria for GD, and PUSM, which had been adapted for this purpose (C.-H. Ko & Yen, 2022).

##### 2.3.2.2 DSM-5 criteria for PUSM/IGD

A semi-structured interview schedule based on Ko (2022) was used to assess the nine DSM-5 criteria for IGD (C.-H. Ko & Yen, 2022), which were adapted for PUSM. A diagnosis required meeting at least five of the nine criteria (American Psychiatric Association, 2013).

##### 2.3.2.3 Mini International Neuropsychiatric interview (MINI)

This structured interview was used to screen for and exclude the presence of co-occurring psychiatric disorders, including major depressive disorder, bipolar I disorder, obsessive–compulsive disorder, anxiety disorders, psychotic disorders, and substance use disorders. The Chinese version of the MINI has demonstrated acceptable reliability and validity (Si Tian-Mei et al., 2009).

##### 2.3.2.4 Clinical Global Impression scale (CGI)

This scale was modified to determine the severity of IG/PUSM. A single CGI question was used: “Considering your total clinical experience with this particular population, how mentally ill is the patient at this time?” The modified scale has a scoring range of 1–7, with higher scores indicating greater severity.

##### 2.3.2.5 Functional impairment

This structured interview was developed by adapting the Brief Gaming Negative Consequence Scale (JuYu Yen et al., 2022; C.-H. Ko et al., 2020) to assess functional impairment due to social media use or gaming. This tool evaluates the negative impact of social media use/gaming on six domains: academic performance, peer relationships, teacher-student relationships, family relationships, health, and safety.

#### 2.3.3 Self-Report scales

##### 2.3.3.1 Bergen social media addiction scale (BSMAS)

The Chinese version of the BSMAS is a 5-point 6-item scale assessing PUSM (Andreassen et al., 2017, Leung et al., 2020). Each item of BSMAS represents a core component of Griffiths’ biopsychosocial model for addiction (Mark Griffiths, 2005). The suggested cut-off score for BSMAS is 24 (Luo et al., 2021). In this study, the Cronbach’s α was 0.86.

##### 2.3.3.2 Nine-item Internet gaming disorder scale–short-form (IGDS-SF9)

The Chinese version of the IGDS9-SF is a 5-point 9-item scale based on DSM-5 criteria assessing IGD (Leung et al., 2020, Pontes and Griffiths, 2015). The suggested cut-off score for IGDS9-SF is 32 (Lixia Qin, 2020). In this study, the Cronbach’s α was 0.90.

##### 2.3.3.3 Validation variables

To test the validity of the clinical diagnoses of PUSM and IGD, two key external criteria were used. Adverse mood (e.g., depression) and impulsivity traits have been consistently identified as correlates of addictive states (Brand et al., 2016, Müller et al., 2019). The 9-item Patient Health Questionnaire (Kroenke et al., 2001, Zhang et al., 2013) (PHQ-9) and the Brief Barratt Impulsiveness Scale (Luo et al., 2020, Morean et al., 2014)(BBIS) were therefore adopted. The Chinese versions of these scales have shown good reliability and validity. In this study, the internal consistency reliability was 0.73 for the BBIS and 0.78 for the PHQ-9.

### 2.4 Statistical methods

This study uses the clinical diagnosis as the “gold standard”. By comparing and analyzing the scores from external scales such as PHQ-9 and BBIS, the structural validity and criterion-related validity of the clinical assessment tool for PUSM/GD were validated.

The diagnostic performance of the IGD-11 and DSM-5 criteria was then rigorously evaluated. Key diagnostic metrics were established, including sensitivity (the rate of identifying true positive cases) and specificivity (the rate of correctly identifying true negative cases). Furthermore, to gauge the practical utility of these criteria, the Positive Predictive Value (PPV) and Negative Predictive Value (NPV) were calculated. The Diagnostic Accuracy (DA), representing the overall ability of the criteria to correctly distinguish between cases and non-cases, was also determined.

To further investigate the clinical consistency between PUSM and GD, this study conducted a comparative analysis. This analysis focused on examining the similarities and differences between the two disorders in terms of diagnostic criteria, functional impairment, and clinical severity.

## 3 Results

As shown in Table 1, the five study groups (PUSM, GD, RSMU, RG, and Control) were successfully matched on key demographic variables, with no significant differences in gender or age.

**Table 1:** The demographic data and gaming/social media use behavior of SMD, GD, RSMU, RG, and control group.

	SMD (N = 81)	GD (N = 81)	RSMU (N = 81)	RG (N = 81)	Control (N = 81)		
Variable	N (%)/M (SD)	N (%)/M (SD)	N (%)/M (SD)	N (%)/M (SD)	N (%)/M (SD)	χ^2^/F	Post Hoc
Gender							
Male	48 (59.26)	48 (59.26)	48 (59.26)	48 (59.26)	48 (59.26)	−	−
Female	33 (40.74)	33 (40.74)	33 (40.74)	33 (40.74)	33 (40.74)		
Age	16.86 (1.47)	17.04 (1.31)	17.02 (1.43)	17.04 (1.38)	17.01 (1.40)	0.23	SMD = GD = RSMU = RG = Conrol
BBIS	25.16 (2.48)	25.32 (2.97)	21.53 (2.83)	21.75 (2.33)	16.00 (5.22)	104.09^***^	SMD = GD > RSMU = RG > Control
PHQ-9	8.78 (2.51)	8.72 (2.72)	6.63 (2.42)	6.32 (2.71)	3.82 (2.38)	54.24^***^	SMD = GD > RSMU = RG > Control
BSMAS	27.54 (2.50)	13.15 (2.96)	19.47 (2.77)	13.20 (2.82)	14.12 (3.23)	379.52^***^	SMD > RSMU > RG = GD = Control
IGDS-SF9	19.32 (3.98)	38.37 (8.33)	16.63 (4.67)	19.20 (4.12)	15.81 (3.57)	129.44^***^	GD > SMD = RG > RSMU = Control
Weekly social media use (hours)	49.68 (12.26)	14.72 (7.10)	19.38 (4.74)	12.84 (4.92)	13.15 (6.52)	345.29^***^	SMD > RSMU > GD = RG = Control
Weekly game play (hours)	9.25 (7.96)	50.60 (15.73)	11.46 (8.02)	21.35 (5.27)	7.60 (7.32)	284.22^***^	GD > RG > RSMU > SMD = Control

Note. ^***^p < 0.001.

SMD: social media disorder; GD: gaming disorder; RSMU: regular social media use; RG: regular gaming.

BBIS: brief Barratt impulsiveness scale; PHQ-9: 9-item patient health questionnaire; BSMAS: Bergen social media addiction scale; IGDS-SF9: Internet Gaming Disorder Scale-Short Form.

### 3.1 Validity of psychiatrists’ diagnosis based on ICD-11 criteria

To assess the diagnostic efficacy of the adapted ICD-11-GD criteria, we compared the scores of the BBIS and PHQ-9 scales across the different participant groups. Both the PUSM and GD groups exhibited significantly higher levels of impulsivity (BBIS) and depressive symptoms (PHQ-9) compared to the regular use (RSMU, RG) and control groups, with PUSM and GD showing similar and severe profiles (PUSM = GD > RSMU = RG > Control).

Consistent with group definitions, the PUSM group had a significantly higher score on the BSMAS than all other groups, while the GD group showed the highest score on the IGDS-SF9. Regarding behavioral patterns, the PUSM group reported significantly longer weekly social media use (49.68 h) than all other groups, whereas the GD group reported significantly longer weekly gameplay (50.60 h).

### 3.2 Diagnostic performance of the ICD-11 and DSM-5 criteria

Using a clinical diagnosis based on ICD-11-GD as the gold standard, the applicability of the DSM-5-IGD criteria in the context of PUSM was examined. The four core diagnostic criteria of ICD-11—“loss of control,” “increased priority,” “excessive use despite negative consequences,” and “functional impairment”—demonstrated 100% sensitivity for both GD and PUSM, along with good specificity (Tables 2 and 3). Among the nine DSM-5 criteria, most showed good diagnostic performance (sensitivity, specificity, and diagnostic accuracy all > 80%), with “deception” being the notable exceptions. The “deception” criterion exhibited the lowest diagnostic performance for both disorders, with a sensitivity of 70.37% and 71.60% (for PUSM and GD, respectively), and specificities of 82.10% and 72.22%, yielding diagnostic accuracies of 78.19% and 72.02%. This finding is similar for both PUSM and GD, suggesting that “deception” may have low utility in the diagnosis of behavioral addictions.

**Table 2:** The diagnostic accuracy of ICD-11 and DSM-5 criteria for social media disorder.

Variable	SMD (N = 81) N (%)	RSMU (N = 81) N (%)	Control (N = 81) N (%)	χ^2^	Sensitivity (%)	Specificity (%)	Diagnostic accuracy (%)
Loss of control							
Yes	81 (71.05)	22 (19.30)	10 (9.65)	137.48^***^	100	80.25	86.83
No	0 (0.00)	59 (45.74)	71 (54.26)				

Increased priority in social media use							
Yes	81 (76.41%)	20 (18.87%)	5 (4.71%)	159.68^***^	100	84.57	89.71
No	0 (0.00%)	61 (44.52%)	76 (55.48%)				

Excessive social media use despite negative consequence							
Yes	81 (66.39%)	20 (29.51%)	5 (4.10%)	159.68^***^	100	84.57	89.71
No	0 (0.00%)	61 (37.19%)	76 (62.81%)				

Functional impairment							
Yes	81 (83.51%)	12 (12.37%)	4 (4.12%)	179.77^***^	100	90.12	93.42
No	0 (0.00%)	69 (47.26%)	77 (52.74%)				

Preoccupation							
Yes	75 (65.22%)	32 (27.83%)	8 (6.95%)	94.31^***^	92.59	75.31	80.07
No	6 (4.69%)	49 (38.28%)	73 (57.03%)				

Tolerance							
Yes	73 (69.52%)	27 (25.71%)	5 (4.76%)	108.97^***^	90.12	80.25	83.54
No	8 (5.80%)	54 (39.13%)	76 (55.07%)				

Withdrawal							
Yes	74 (83.15%)	14 (15.73%)	1 (1.12%)	156.81^***^	91.36	90.74	90.95
No	7 (4.55%)	67 (43.51%)	80 (51.95%)				

Give up other activites							
Yes	75 (82.41%)	13 (14.29%)	3 (3.30%)	160.81^***^	92.59	90.12	90.95
No	6 (3.95%)	68 (44.74%)	78 (51.31%)				

Deception							
Yes	57 (66.28%)	26 (30.23%)	3 (3.49%)	57.89^***^	70.37	82.10	78.19
No	24 (15.29%)	55 (35.03%)	78 (49.68%)				

Escape							
Yes	69 (80.23%)	14 (16.28%)	3 (3.49%)	123.43^***^	85.19	89.51	91.45
No	12 (7.64%)	67 (42.68%)	78 (49.68%)				

SMD in DSM-5							
Yes	79 (85.87)	13 (14.13)	0 (0.00)	183.88^***^	97.53	91.98	93.83
No	2 (1.32)	68 (45.03)	81 (53.64)				

Clinical Global Impression							
1 Normal	0 (0.00)	61 (42.96)	81 (57.04)	271.43^***^			
2 Excessive use	0 (0.00)	11 (100.00)	0 (0.00)				
3 Mild	0 (0.00)	8 (100.00)	0 (0.00)				
4 Moderate	28 (96.55)	1 (3.45)	0 (0.00)				
5 Marked	38 (100.00)	0 (0.00)	0 (0.00)				
6 Severe	15 (100.00)	0 (0.00)	0 (0.00)				

Note.^***^p < 0.001.

SMD: social media disorder; RSMU: regular social media use.

SMD in DSM-5: Social media disorder diagnosis based on criteria of DSM-5 for internet gaming disorder.

Diagnostic accuracy: (True positive + True negative)/All.

**Table 3:** The diagnostic accuracy of ICD-11 and DSM-5 criteria for gaming disorder.

Variable	GD (N = 81) N (%)	RG (N = 81) N (%)	Control (N = 81) N (%)	χ^2^	Sensitivity (%)	Specificity (%)	Diagnostic accuracy (%)
Loss of control							
Yes	81 (71.05)	22 (19.30)	10 (9.65)	137.48^***^	100	80.25	86.83
No	0 (0.00)	59 (45.74)	71 (54.26)				

Increased priority in gaming							
Yes	81 (73.64)	25 (22.73)	4 (3.64)	146.90^***^	100	82.1	88.07
No	0 (0.00)	56 (42.11)	77 (57.89)				

Excessive gaming despite negative consequence							
Yes	81 (71.68)	27 (23.89)	5 (4.42)	139.77^***^	100	80.25	86.83
No	0 (0.00)	54 (41.54)	76 (58.46)				

Functional impairment							
Yes	81 (83.51)	11 (11.34)	5 (5.15)	182.87^***^	100	90.12	93.42
No	0 (0.00)	70 (47.95)	76 (52.05)				

Preoccupation							
Yes	72 (62.07)	33 (28.45)	11 (9.48)	82.49^***^	88.89	72.22	77.78
No	9 (7.14)	47 (37.30)	70 (55.56)				

Tolerance							
Yes	73 (70.87)	25 (24.27)	5 (4.85)	113.37^***^	90.12	81.48	84.36
No	8 (5.71)	56 (40.00)	76 (54.29)				

Withdrawal							
Yes	70 (87.50)	9 (11.25)	1 (1.25)	157.46^***^	86.42	93.83	91.36
No	11 (6.75)	72 (44.17)	80 (49.08)				

Give up other activites							
Yes	72 (79.12)	17 (18.68)	2 (2.20)	137.25^***^	88.89	88.27	88.48
No	9 (5.92)	64 (42.11)	79 (51.97)				

Deception							
Yes	58 (56.31)	38 (36.89)	7 (6.80)	42.47^***^	71.60	72.22	72.02
No	23 (16.43)	43 (30.71)	74 (52.86)				

Escape							
Yes	73 (72.28)	22 (21.78)	6 (5.94)	117.97^***^	90.12	82.1	84.77
No	8 (5.67)	58 (41.13)	75 (53.19)				

IGD in DSM-5							
Yes	80 (87.91)	11 (12.09)	0 (0.00)	183.88^***^	98.77	93.21	95.06
No	1 (0.66)	70 (46.05)	81 (53.29)				

Clinical Global Impression							
1 Normal	0 (0.00)	70 (46.36)	81 (53.64)	260.70^***^			
2 Excessive gaming	0 (0.00)	11 (100.00)	0 (0.00)				
3 Mild	0 (0.00)	0 (0.00)	0 (0.00)				
4 Moderate	29 (100.00)	0 (0.00)	0 (0.00)				
5 Marked	36 (100.00)	0 (0.00)	0 (0.00)				
6 Severe	16 (100.00)	0 (0.00)	0 (0.00)				

Note.^***^p < 0.001.

GD: gaming disorder; RG: regular gaming. IGD in DSM-5: Internet gaming disorder diagnosis based on criteria of *Diagnostic and Statistical Manual of Mental Disorders, Fifth Edition.*

CGI: Clinical Global Impression Scale.

Diagnostic accuracy: (True positive + True negative)/All.

### 3.3 Association between the ICD-11-GD and DSM-5-IGD criteria

We further analyzed the consistency between the ICD-11-GD and DSM-5-IGD diagnostic criteria. For PUSM, the DSM-5 criteria demonstrated high diagnostic accuracy (93.83%) in identifying patients diagnosed with ICD-11 (Table 2). A direct comparison revealed that 79 participants were diagnosed with PUSM by both sets of criteria. While 81 participants met ICD-11 criteria, 79 were identified by DSM-5. Conversely, 13 participants identified by the DSM-5 criteria did not meet the ICD-11 criteria.

A similar pattern of high consistency was observed for GD, where the DSM-5 criteria showed 95.06% diagnostic accuracy against the ICD-11 standard (Table 3). Discordant cases highlighted the ICD-11 criteria’s higher threshold: 91 participants met DSM-5 criteria, but only 80 were classified as GD by ICD-11.

### 3.4 Clinical profiles of GD and PUSM

To examine their potential classification within a unified behavioral addiction framework, we systematically compared GD and PUSM across symptom profiles, functional impairment, and clinical severity. As shown in Table 4, no significant differences were found in the rates of meeting any of the four ICD-11 criteria and nine DSM-5 criteria between the PUSM and GD groups. This indicates that the core behavioral symptoms are similarly applicable to both PUSM and GD, suggesting a shared underlying addictive mechanism.

**Table 4:** Comparison of the diagnostic criteria in patients with Gaming Disorder (GD) and patients with Social Media Disorder (SMD).

Variable	SMD (N = 81) N (%)	GD (N = 81) N (%)	χ^2^	*P*-value
loss control	81 (100)	81 (100)		
Increased priority	81 (100)	81 (100)		
Excessive gaming	81 (100)	81 (100)		
Funcional impairment	81 (100)	81 (100)		
Preoccupation	75 (92.59)	72 (88.89)	0.66	0.42
Tolerance	73 (90.12)	73 (90.12)	0.00	1.0
Withdrawal	74 (91.36)	70 (86.42)	1.0	0.32
Give up other activites	75 (92.59)	72 (88.89)	0.66	0.42
Deception	57 (70.37)	58 (71.60)	0.03	0.86
Escape	69 (85.19)	73 (90.12)	0.91	0.34

There were no significant differences in the functional impairment domains between the PUSM and GD groups (Table 5). All participants in both the PUSM and GD groups reported severe impairment in academic performance. Regarding interpersonal relationships, family relationships were most frequently reported as being significantly impaired, followed by teacher-student relationships, and then peer relationships. Health problems resulting from addictive social media use and gaming are widespread; however, there are relatively few reports documenting the increased risks associated with these behaviors.

**Table 5:** The functional impairment and Clinical Severity to social media disorder (SMD) and gaming disorder (GD).

Variable	SMD (N = 81) N (%)	GD (N = 81) N (%)	χ^2^	*P*-value
Function impairment				
Academic performance				
Yes	81 (100.00)	81 (100.00)		
No	0 (0.00)	0 (0.00)		

Peer relationships				
Yes	9 (40.91)	13 (59.09)	0.84	0.36
No	72 (51.43)	68 (48.57)		

Student-teacher relationships				
Yes	39 (46.43)	45 (53.57)	0.89	0.35
No	42 (53.85)	36 (46.15)		

Family relationships				
Yes	81 (100.00)	81 (100.00)		
No	0 (0.00)	0 (0.00)		

Health problems				
Yes	26 (46.43)	30 (53.57)	0.44	0.51
No	55 (51.89)	51 (48.11)		
Near danger				
Yes	1 (16.67)	5 (83.33)	2.77	0.09
No	80 (51.28)	76 (48.72)		

Clinical Global Impression				
4 Moderate	28 (49.12)	29 (50.80)	0.10	0.95
5 Marked	38 (51.35)	36 (48.64)		
6 Severe	15 (48.39)	16 (51.61)		

The CGI scale results indicated a clear clinical need for intervention, with all patients in both the GD and PUSM groups being assessed as “ill” (CGI scores of ≥ 4). A comparison of clinical severity between GD and PUSM revealed no significant differences in the distribution of mild, marked, and severe cases.

## 4 Discussion

Based on clinical interviews, this study systematically evaluated the applicability and diagnostic efficacy of the DSM-5 diagnostic criteria for IGD when applied to PUSM. The results indicated that, for diagnosing PUSM, with the exception of the “deception” criterion, the diagnostic accuracy of the remaining eight criteria all exceeded 80%. This demonstrates that the criteria system of the DSM-5 for diagnosing IGD possesses high clinical representativeness and applicability when translated to the context of PUSM.

### 4.1 Validation of the DSM-5 IGD diagnostic criteria for PUSM

The criteria of “loss of control,” “continuation despite problems,” and “impaired function” have been widely recognized as necessary for the diagnosis of IGD (Billieux et al., 2017, Carbonell, 2017, Kuss et al., 2017). The excellent diagnostic accuracy of these criteria in this study further suggests that they have important clinical significance for diagnosing individuals with PUSM. The findings also support the validity of the IGD-11 model for diagnosing PUSM. While van den Eijnden (van den Eijnden et al., 2016) reported rather low sensitivities (<65%) for “continuation despite problems” and “impaired function” for diagnosing PUSM, contrasting with our findings. This discrepancy likely stems from fundamental differences in sample characteristics. Community-based studies include numerous users whose problems do not meet the clinical threshold, diluting the sensitivity of these criteria. In contrast, the clinical sample in this study comprised help-seeking patients for whom functional impairment was a core reason for seeking help, inevitably leading to high sensitivity of this criterion in case identification. This finding demonstrate the excellent discriminatory power of the DSM-5 criteria for identifying severe social media use disorder when applied in clinical settings.

The “preoccupation” criterion exhibited relatively low specificity in this study, consistent with prior findings in IGD (Király et al., 2017, Pontes et al., 2019). Our previous study also showed a low predictive value of this criterion for the diagnosis of PUSM (Luo et al., 2021). This prompts critical reflection on its construct. This criterion measures persistent cognitive preoccupation with social media. However, this study found that the “give up other activities” criterion, reflecting behavioral salience, demonstrated higher specificity and diagnostic accuracy. This aligns with the theory proposed by Kardefelt-Winther, which emphasizes the essential distinction between “high engagement” and “pathological addiction” (Griffiths et al., 2016, Kardefelt-Winther et al., 2017, Kardefelt-Winther, 2014, Kardefelt-Winther, 2015). Purely cognitive preoccupation may stem from interest in social media content, falling within the realm of high engagement. It is only when this cognition translates into the systematic sacrifice of other important life activities (e.g., studies, social interactions, or sports) that it is more likely to indicate a clinically significant disorder.

Although “tolerance” and “withdrawal” have been regarded as biological concepts and criticized for their use as criteria for behavioural addiction (Griffiths et al., 2016, Starcevic, 2017), the excellent diagnostic performance suggests that these two criteria are strong indicators of PUSM. An increasing number of recent neurophysiological studies have provided compelling evidence for similar changes in the brain between behavioural addictions and substance-related addictions (Burleigh et al., 2020, Kuss and Griffiths, 2012).

The “escape” criterion demonstrated high specificity in diagnosing PUSM (C. H. Ko et al., 2014; C.-H. Ko et al., 2020), similar to our previous findings in general adolescent populations. Its high specificity can be explained from the perspective of behavioral motivation: for regular users, usage often stems from positive reinforcement motives like seeking pleasure or social connection; whereas for addicted individuals, the core function of use shifts to a negative reinforcement mechanism of coping with negative emotions and escaping real-world problems.

The “deception” criterion showed the lowest diagnostic accuracy in this study, consistent with related IGD findings (C. H. Ko et al., 2014, Müller et al., 2019). This result suggests limited inherent validity of this criterion as a diagnostic indicator for behavioral addiction. It appears to be influenced more by confounding factors such as external environment, cultural norms, and family interaction patterns, rather than purely reflecting addictive pathology.

These findings not only validate the applicability of these diagnostic criteria derived from IGD in the context of social media use, but more importantly, provide direct empirical evidence for further conceptualizing PSMU as a clinically significant behavioral addiction. This moves beyond mere symptom description, revealing its shared core psychopathological basis with gaming disorder, namely, the breakdown of behavioral control and the resulting functional impairment across multiple domains. Therefore, this research adds important validity evidence for its potential future inclusion in formal diagnostic classification systems, such as revised editions of the ICD-11 or DSM-5.

### 4.2 Diagnostic CONCORDANCE AND THRESHOLDS BETWeen ICD and 11 and DSM-5

This study, through a direct comparison of the ICD-11 GD and DSM-5 IGD diagnostic criteria, reveals a high level of consistency between the two systems in identifying clinical cases, which is in accordance with findings from studies on IGD (JuYu Yen et al., 2022)**.** For PUSM, the DSM-5 criteria demonstrated a diagnostic accuracy of 95.47%, with the vast majority of patients (78/81) receiving a concurrent diagnosis under both frameworks. This finding strongly indicates that despite differences in conceptual frameworks and diagnostic thresholds, both sets of criteria are equally effective in capturing core, clinically significant cases of gaming disorder.

However, the subtle discrepancies are particularly revealing. A consistent pattern was observed across both gaming and Problematic Social Media Usages, indicating that the ICD-11 criteria employ a stricter diagnostic threshold, aligning with previous research (JuYu Yen et al., 2022; C.-H. Ko et al., 2020). This pattern suggests that while the DSM-5 criteria, with their broader inclusion scope, may possess higher sensitivity, they might also capture some “problematic users” whose condition does not reach the threshold of clinical severity. In contrast, the ICD-11’s focus on core behavioral patterns—“loss of control,” “increasing priority,” “continuation despite negative consequences,” and clear functional impairment—likely results in higher diagnostic specificity (Billieux et al., 2017, Carbonell, 2017, Kuss et al., 2017). This approach better ensures that the identified cases warrant clinical attention due to their severity and the urgency of intervention.

### 4.3 A unified framework with shared functional impairment

Direct comparison between PUSM and GD patients across diagnostic criteria revealed no statistically significant differences for all criteria. Clinical Global Impression scale scores also showed no significant difference between groups, with all patients rated at least “moderate” in severity. This discovery provides robust evidence for the “unified framework of behavioral addictions” hypothesis, suggesting that despite differences in their outward manifestations, addictions to both social media and gaming stem from a shared underlying mechanism. Differences between addictive behaviors primarily lie in the specific content of the behavioral vehicle, while loss of control over the behavior and resulting functional impairment are commonalities.

The study also found high homogeneity in social functional impairment between PUSM and GD, commonly manifested in the two key developmental pillars for adolescents: academic performance and family relationships. Academic decline, often simply attributed to time displacement, may underlie complex neurocognitive hijacking. Widespread impairment in family relationships reveals the relational toxicity of behavioral addiction. Parental concern and intervention are perceived as “control,” triggering adolescent resistance and avoidance, while adolescent withdrawal and emotional disconnection exacerbate parental anxiety and ineffective parenting. This interaction pattern erodes family emotional bonds, transforming the family from a potential stress buffer into a primary stressor itself. This indicates that interventions should focus not only on the addictive behavior itself (e.g., limiting screen time) but also on repairing specific functional domains.

### 4.4 Limitations

This study has several limitations. First, craving—a core motivational process in addiction—was not directly assessed. This was a strategic choice prioritizing the primary objective of validating the original diagnostic framework’s cross-behavior applicability, while also considering the conceptual overlap between “preoccupation” and “craving.” Second, the sample inclusion and exclusion criteria limit the generalizability of the findings. Patients with PUSM in broader clinical settings may present with more complex clinical profiles. Third, the “deception” criterion requires further cross-cultural, multi-sample, multi-method empirical research for continuous refinement. Fourth, While the current findings primarily reveal similarities between GD and PSMU, future research would benefit from more robustly considering and elaborating on the psychological and psychopathological distinctiveness of PSMU compared to other forms of problematic internet use, as well as the differences in the nature of social media use (active versus passive, scrolling, content creation, relational interaction) and their underlying motivations. Such efforts would contribute to a more comprehensive understanding of PSMU and help establish a better framework for its diagnosis and intervention. Fifth, the use of clinician-rated instruments (such as the CGI scale) to determine the severity level of participants, while reflecting real-world diagnostic practices, also introduces a noteworthy potential source of variability. Lastly, the assessment tools employed, including measurement methods such as semi-structured interviews and self-report scales, are subject to known biases. Moreover, the Bergen Social Media Addiction Scale used in this study is a unidimensional tool and may not fully capture the complexity of the construct, as emerging evidence suggests that a two-factor model distinguishing between core and peripheral symptoms could provide a more nuanced assessment (e.g., Fournier et al., 2023).

## 5 Conclusions

In conclusion, this study validates that the DSM-5 IGD diagnostic criteria demonstrate excellent diagnostic efficacy for PUSM, although the clinical utility of the “deception” criterion requires further validation. The findings support conceptualizing PUSM as a clinical entity that is homogeneous with IGD in its core symptomatology. Given the current absence of official diagnostic criteria for PUSM, this validated criteria set can serve as a practical assessment toolkit for clinicians and researchers to systematically evaluate disorder severity and guide the development of intervention strategies.

## 6 Ethics

Informed consent was obtained from all participants, while parents’ permission was also obtained for those less than 18 years of age. The procedures were carried out in accordance with the Declaration of Helsinki. Ethical approval was also attained from the local ethics committee (No. 20180112).

## Author contributions

Wenxia Xie, and Tao Luo: contributed in conceptualizing and designing the study, collecting, analysis and interpretation of data, drafting and revising the article, and final approval of the version to be published. Shaoke Cao, Xuelin Chao, Qian Sun, Qing Zou, Chuanjian Liu, Guojiang Wu, Qiaosheng Liu: contributed in collecting, analysis, and interpretation of data, and final approval of the version to be published. Xiaoping Wang, Wei Hao, and Yanhui Liao: contributed in designing and supervising the study.

## CRediT authorship contribution statement

**Wenxia Xie:** Writing – review & editing, Writing – original draft, Methodology, Formal analysis, Data curation, Conceptualization. **Shaoke Cao:** Writing – review & editing, Investigation, Formal analysis, Data curation. **Xuelin Chao:** Writing – review & editing, Investigation, Formal analysis, Data curation. **Qian Sun:** Writing – review & editing, Investigation, Formal analysis, Data curation. **Qing Zou:** Writing – review & editing, Investigation, Formal analysis, Data curation. **Chuanjian Liu:** Writing – review & editing, Investigation, Formal analysis, Data curation. **Guojiang Wu:** Writing – review & editing, Investigation, Formal analysis, Data curation. **Qiaosheng Liu:** Writing – review & editing, Investigation, Formal analysis, Data curation. **Xiaoping Wang:** Supervision, Methodology, Conceptualization. **Wei Hao:** Supervision, Methodology, Conceptualization. **Yanhui Liao:** Supervision, Methodology, Conceptualization. **Tao Luo:** Writing – review & editing, Writing – original draft, Investigation, Formal analysis, Data curation, Conceptualization.

## Funding

This study was supported by grants from the National Natural Science Foundation of China (72264024) to Tao Luo.

## Declaration of competing interest

The authors declare that they have no known competing financial interests or personal relationships that could have appeared to influence the work reported in this paper.

## Data Availability

Data will be made available on request.
